# Supplementary figures and images for: Genomes of Leishmania parasites directly sequenced from patients with visceral leishmaniasis in the Indian subcontinent
Source: PLoS Negl Trop Dis. 2019 Dec 12;13(12):e0007900. doi: 10.1371/journal.pntd.0007900 (PMC6932831; doi:10.1371/journal.pntd.0007900)

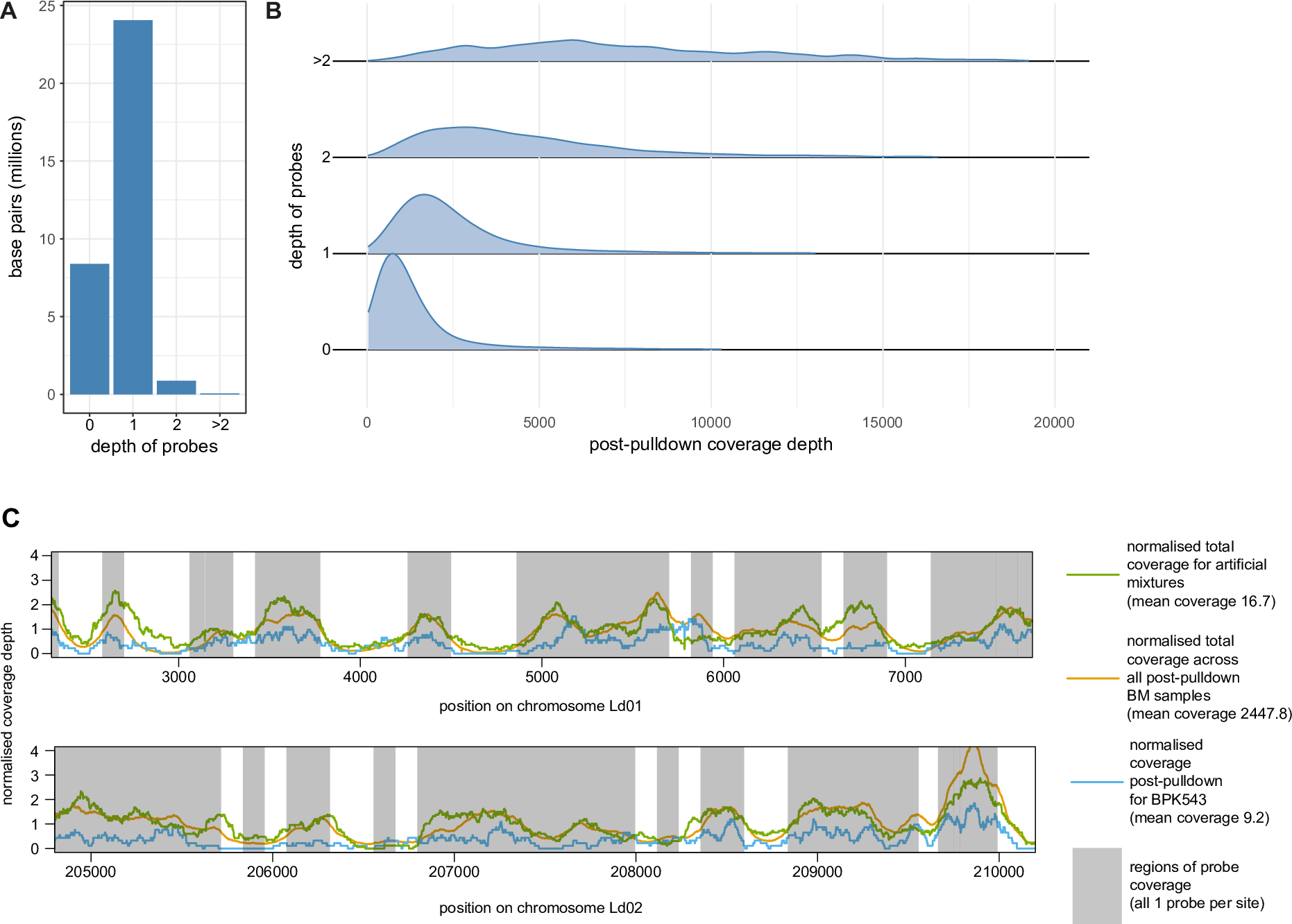

Supplement: S1 Fig — A. Distribution of probes across L. donovani BPK282 reference genome. Most of the reference genome is covered by only a single probe. Some regions are not covered to avoid repetitive regions and regions with homology to mammalian hosts. Changes in the genome assembly subsequent to the bait design and the presence of additional probes to capture L. infantum have led to some regions being covered by multiple probes sequences. B. Much of the variation in depth in coverage in clinical samples is due to the presence of multiple probe sequences. Plots show the total coverage density across all clinical samples for regions of the reference genome covered by 0, 1, 2 or more probe sequences. C. Depth of read coverage in SureSelect enriched samples across two regions of the Leishmania genome. Lines show normalized coverage depth (actual coverage per base pair divided by genome-wide mean coverage) for one clinical sample (BPK543, blue) and the sum for all clinical samples (orange). Grey shading shows regions where SureSelect probes map to the genome. Mean coverage for BPK452 was 9.2 reads, for the summed clinical samples 2447.8. (TIF) [file pntd.0007900.s002.tif]

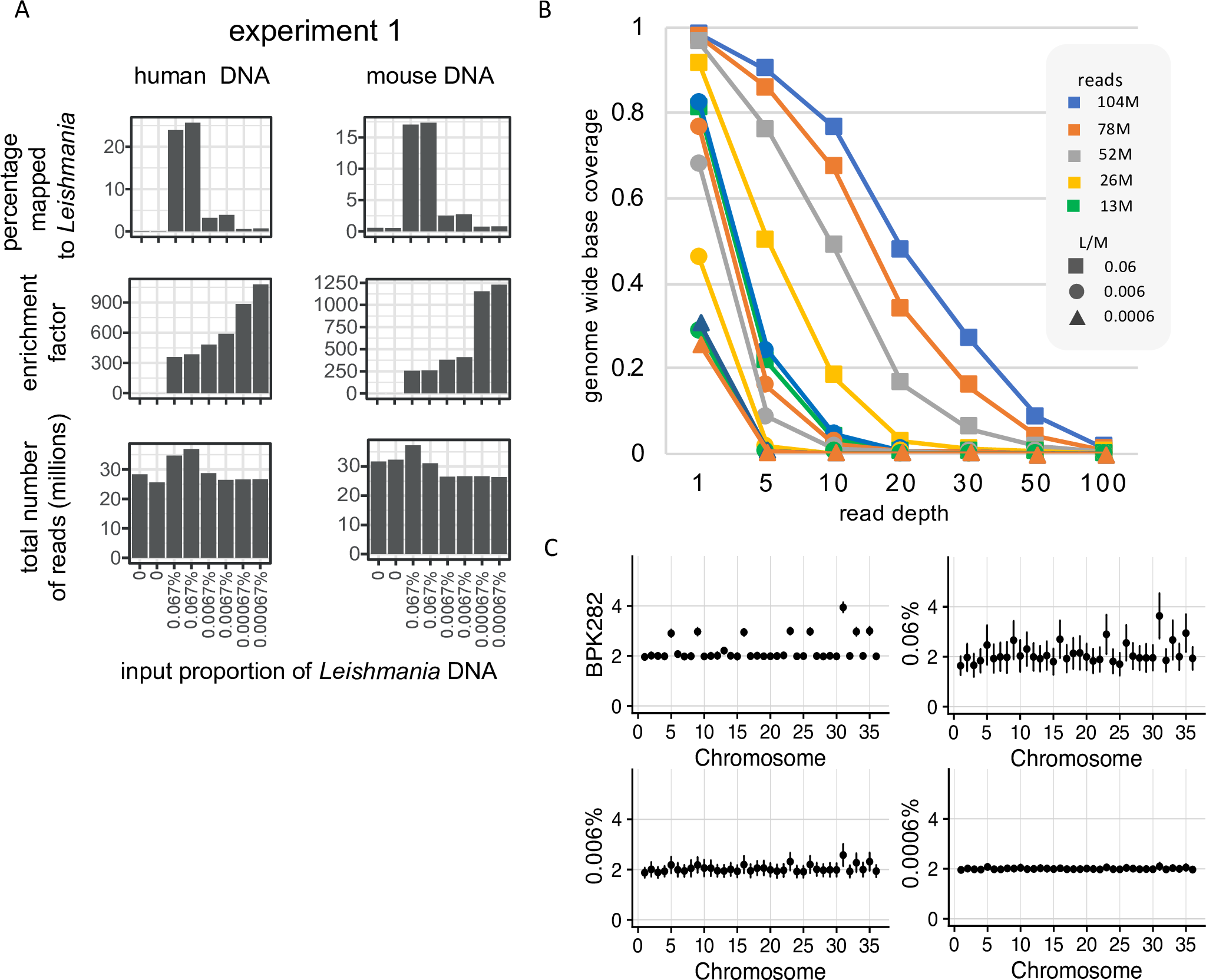

Supplement: S2 Fig — A. Summary statistics for sequencing data from these experiments, showing the total number of reads in each library, the proportion of reads mapping to the L. donovani reference genome, and the enrichment factor, calculated as the ratio of the proportion of reads mapping to L. donovani in the SureSelect libraries to the proportion of Leishmania promastigote DNA included in the pre-pulldown DNA mixtures. B. Evenness of genome coverage. The y-axis shows proportion of bases covered with a minimum number of sequencing reads—read depth along the x-axis— by reads samples from SureSelect sequencing data from artificial mixtures. 104M, 78M, 52M, 26M and 13M indicate the total number of reads (in millions) sampled from each library; 0.06, 0.006 and 0.0006 are the input proportions of Leishmania DNA in percent. For lower Leishmania DNA percentages, the higher read depth did not result in higher genome base coverage. C. Inferred somy from SureSelect on artificial mixtures. BPK282, pure DNA from promastigote; LD06-0006, simulated clinical samples at three Leishmania DNA percentages (0.06%, 0.006% and 0.0006% respectively). Y axis shows normalized somy estimate for each chromosome (x axis). Points show central estimate and bars show one standard deviation around these estimates from four replicates of each Leishmania DNA percentages 0.06%, 0.006%, 0.0006%, respectively. (TIF) [file pntd.0007900.s003.tif]

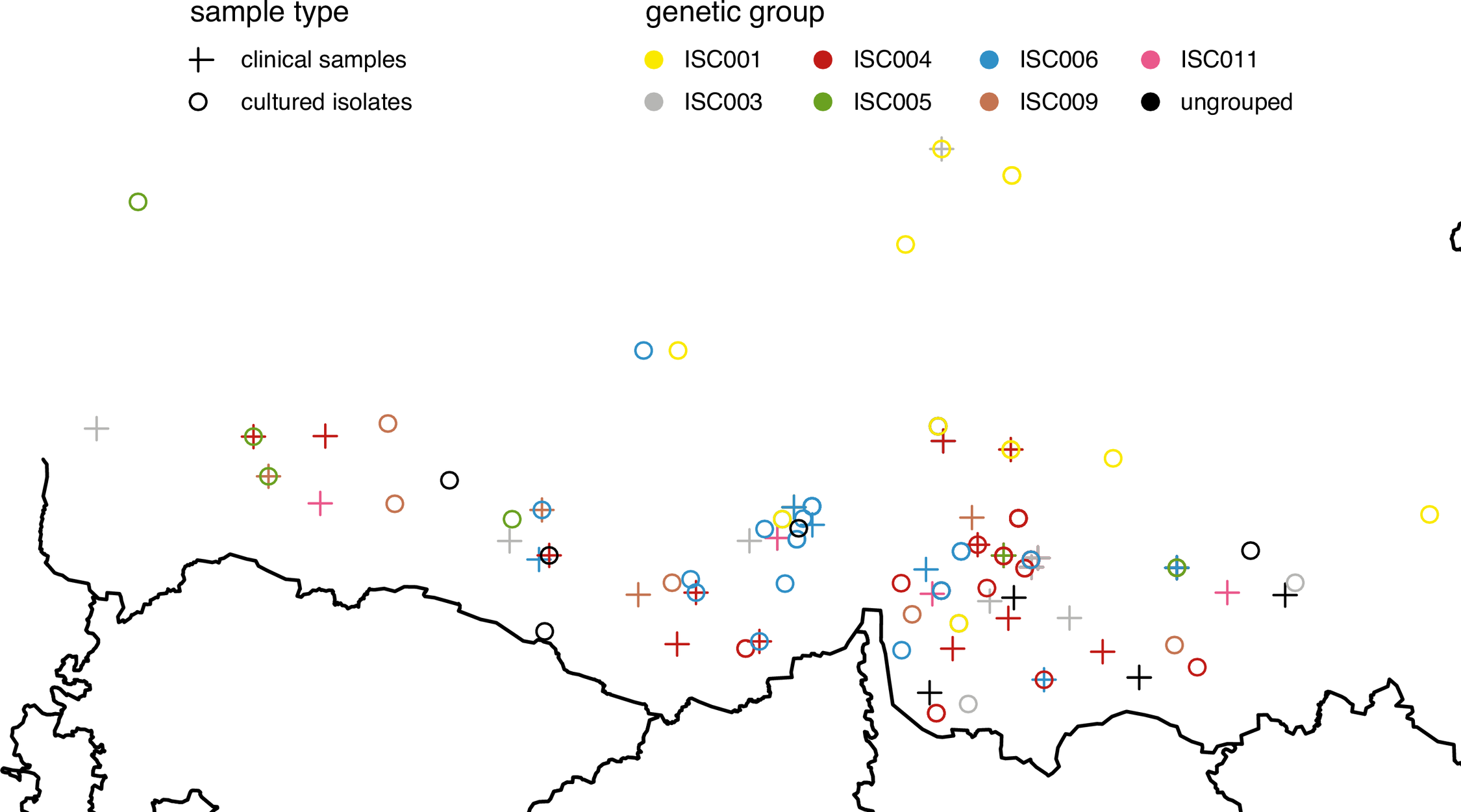

Supplement: S3 Fig — Colours represent different genotype groups defined elsewhere [8]. Circles represent Nepalese promastigote isolates from ref 8, crosses bone marrow and spleen samples. (TIF) [file pntd.0007900.s004.tif]

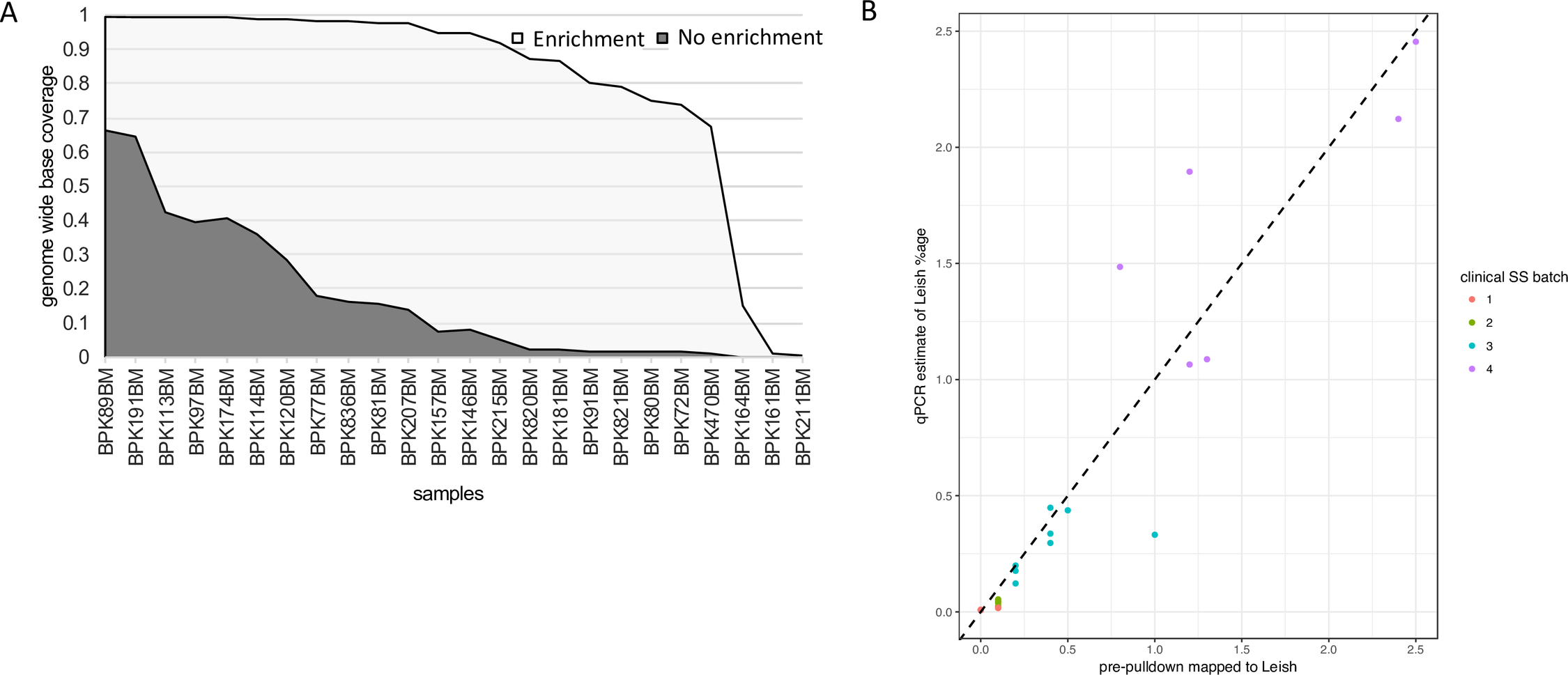

Supplement: S4 Fig — A. Genome sequencing of clinical samples: percentage of the genome covered by more than 1 read, with and without SuSL enrichment. B. Relationship between qPCR estimate of Leishmania DNA concentration (y axis) and the proportion of sequencing reads mapping to the L. donovani reference genome before SureSelect enrichment in clinical samples for which both qPCR and pre-enrichment sequence data are available, confirming accuracy of the qPCR estimates. Batches were formed of samples with similar proportions of Leishmania DNA pre-enrichment. (TIF) [file pntd.0007900.s005.tif]

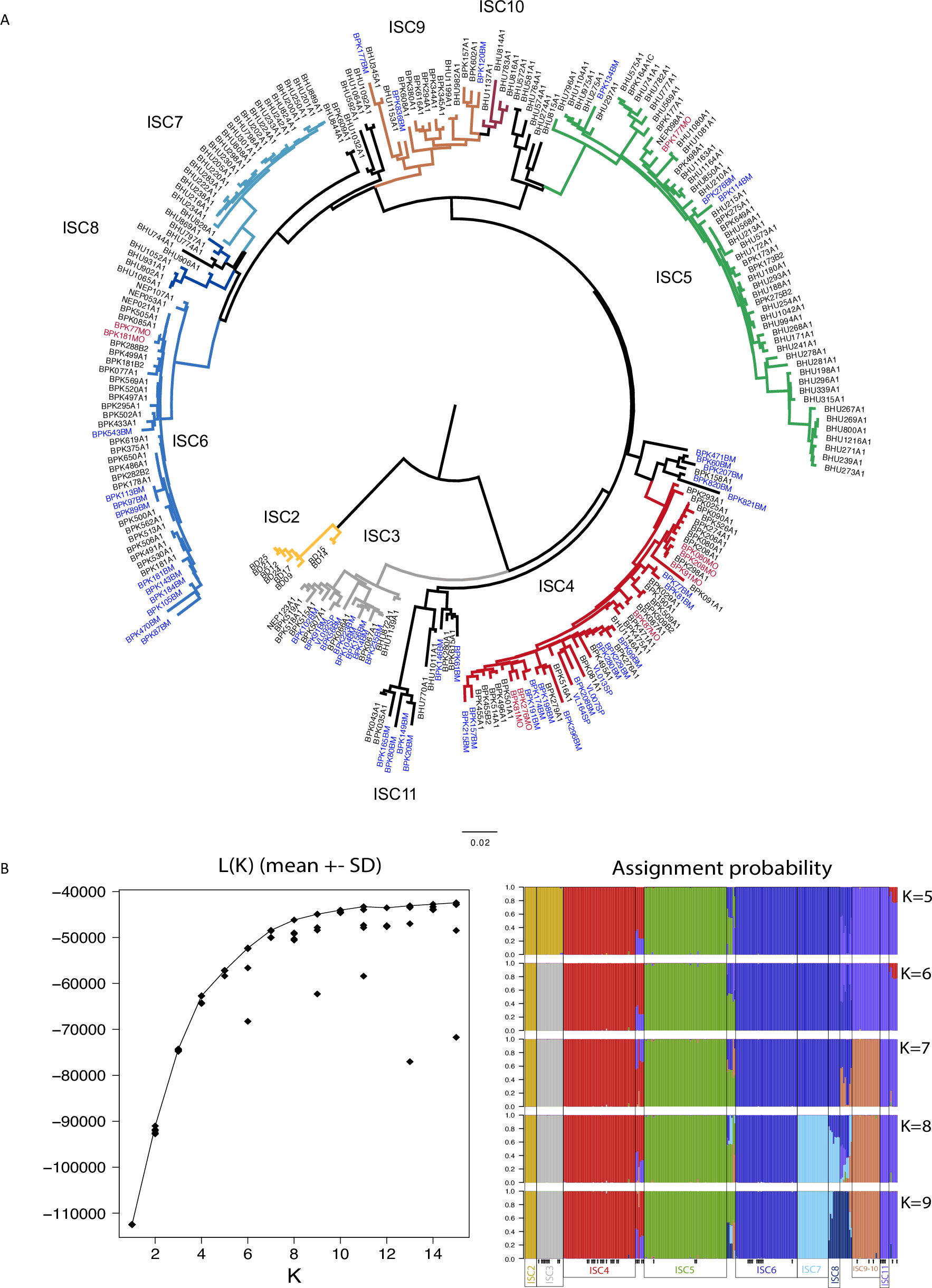

Supplement: S5 Fig — A. Phylogenetic tree (Neighbor-joining) based on bone marrow (BM), spleen (SP), isolates paired to clinical samples (MO) and previously sequenced lines (labelled A1 or B2, ref 8). The tree is identical to the main one (Fig 2A), except that the labels of the samples are indicated. Bone marrow (BM) and spleen (SP) samples are labelled in blue; isolates that are paired to clinical samples are labelled in red, previously sequenced lines are labelled in black. ISC2-ISC10 were sub-populations previously defined (ref 8). See Fig 2A for comments on ISC11. B. Results from STRUCTURE analyses from five replicate runs (2x10^6^ MCMC chains following 106 burn-in steps) under the Admixture model assuming 1–15 K clusters. The plot on the left shows the estimated loglikelihood of all runs for each K cluster. Barplots on the right summarize the assignment probabilities of every L. donovani sequence to each inferred cluster assuming 5, 6, 7, 8 or 9 clusters. Arrows denote the CG samples sequenced in this study. (TIF) [file pntd.0007900.s006.tif]

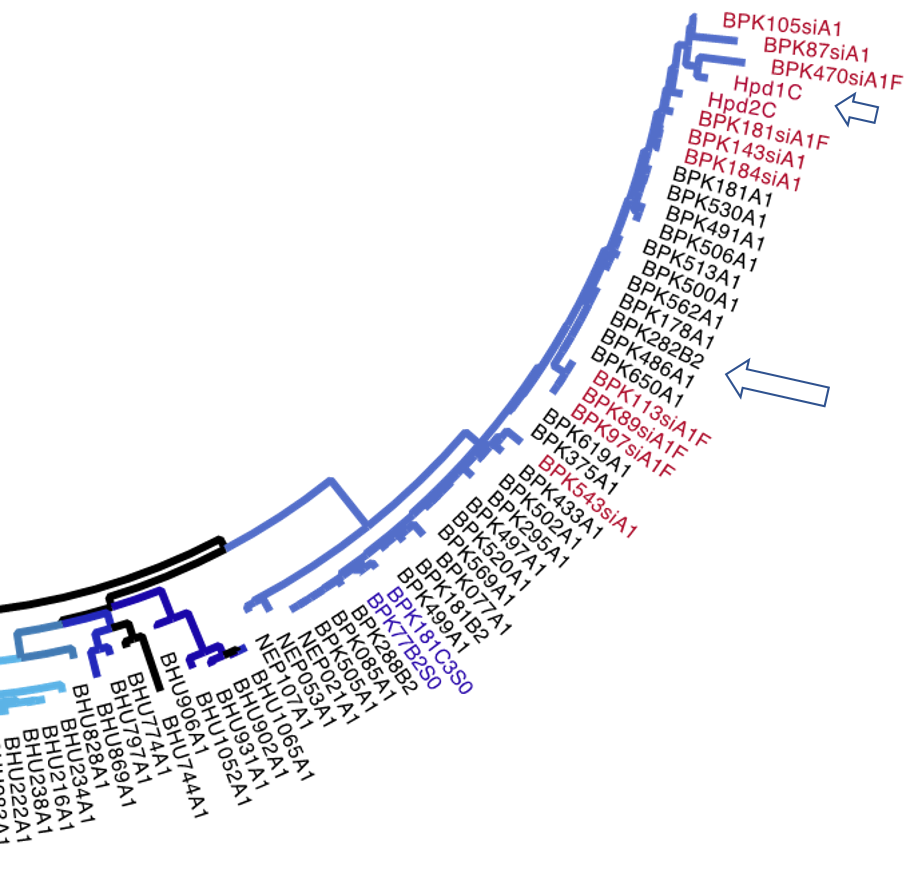

Supplement: S6 Fig — Detail of phylogenetic tree (Neighbor-joining) showing that when adding SuSL-seq data from artificial mixtures (DNA of BPK282 diluted in human DNA, labeled Hpd1C and Hpd2C), they clustered in the ISC6 group with the original BPK282 sequence data (respectively short and long arrows). (TIF) [file pntd.0007900.s007.tif]

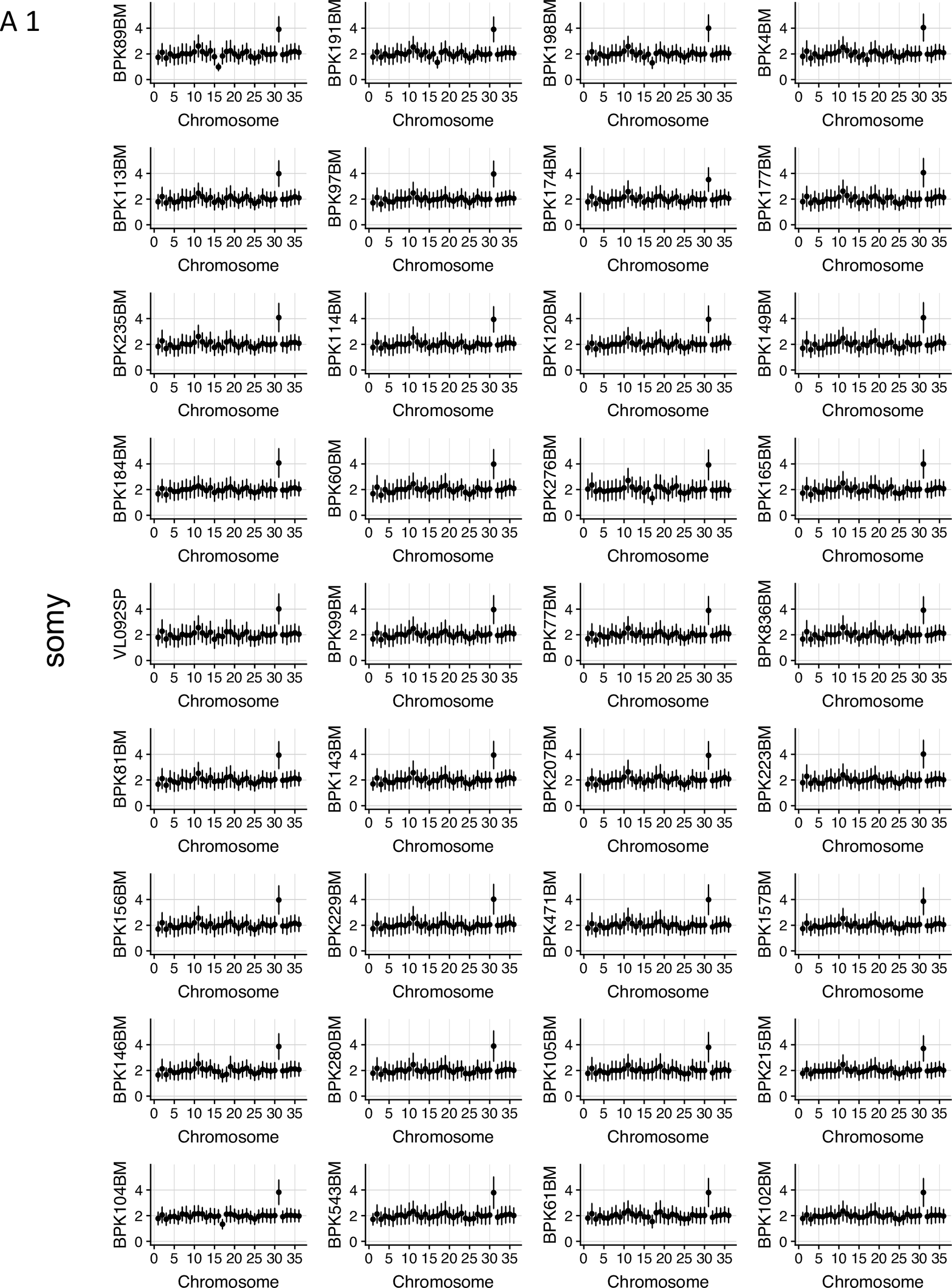

Supplement: S7 Fig — BM = Bone marrow and SP = spleen. The y-axis and x-axis represent somy and chromosome, respectively. S-values and standard deviation error bars are given in black filled circle and vertical line, respectively. The S- and standard deviation values for the bone marrow samples were based on the binned depth method. The samples were sorted in the order of higher genome coverage. (TIF) [file pntd.0007900.s008.tif]

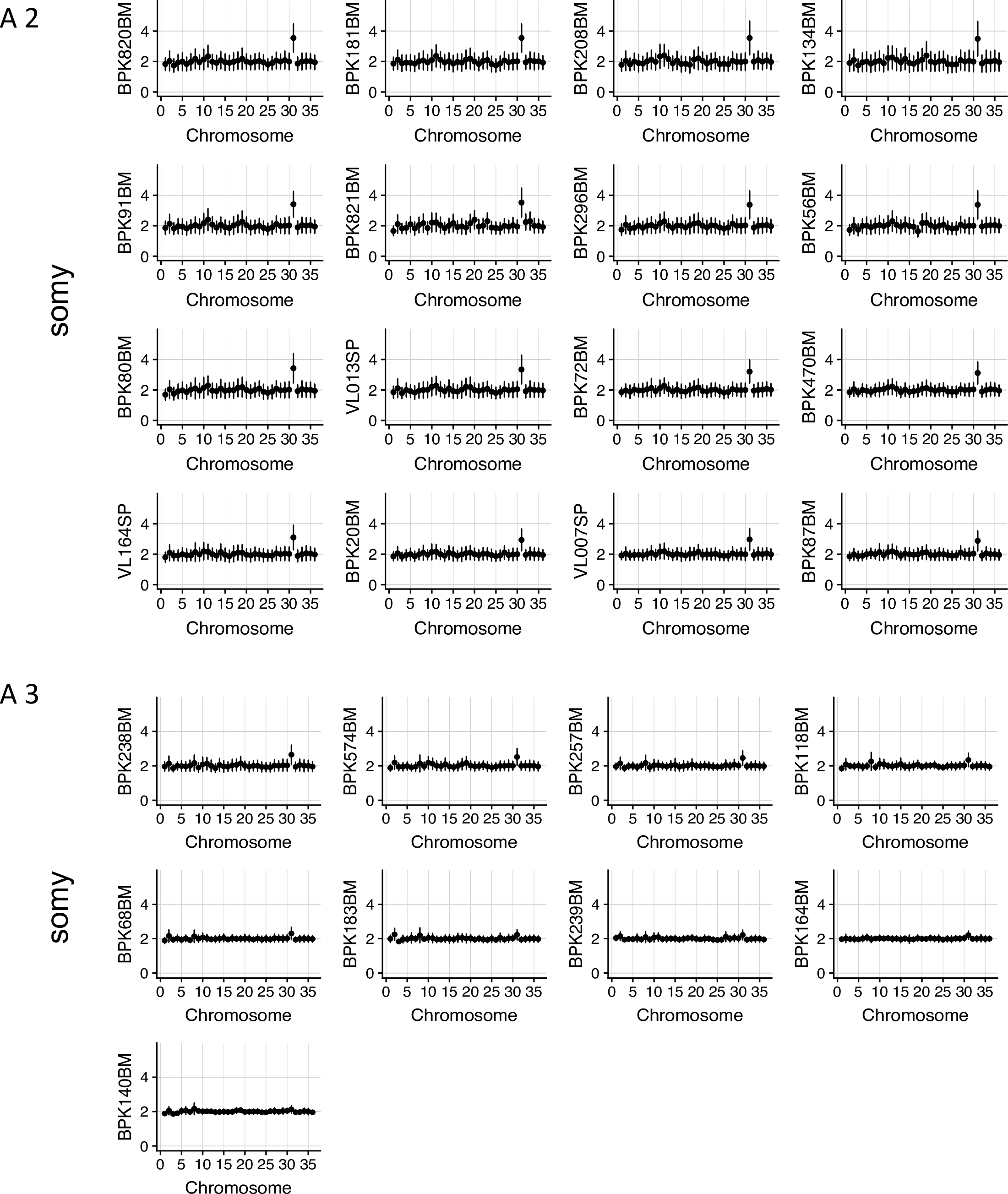

Supplement: S8 Fig — In the lower Leishmania DNA concentration samples, it is still possible to identify higher copy number in chromosome 31. S-values of chromosome 2 and 11 were higher for almost all samples but these higher values were also observed in artificial mixtures of BPK282 where chromosome 2 and 11 were definitively disomic. Close inspection of read depth did not reveal any particular evidences to support their S-values to be higher than 2. (TIF) [file pntd.0007900.s009.tif]

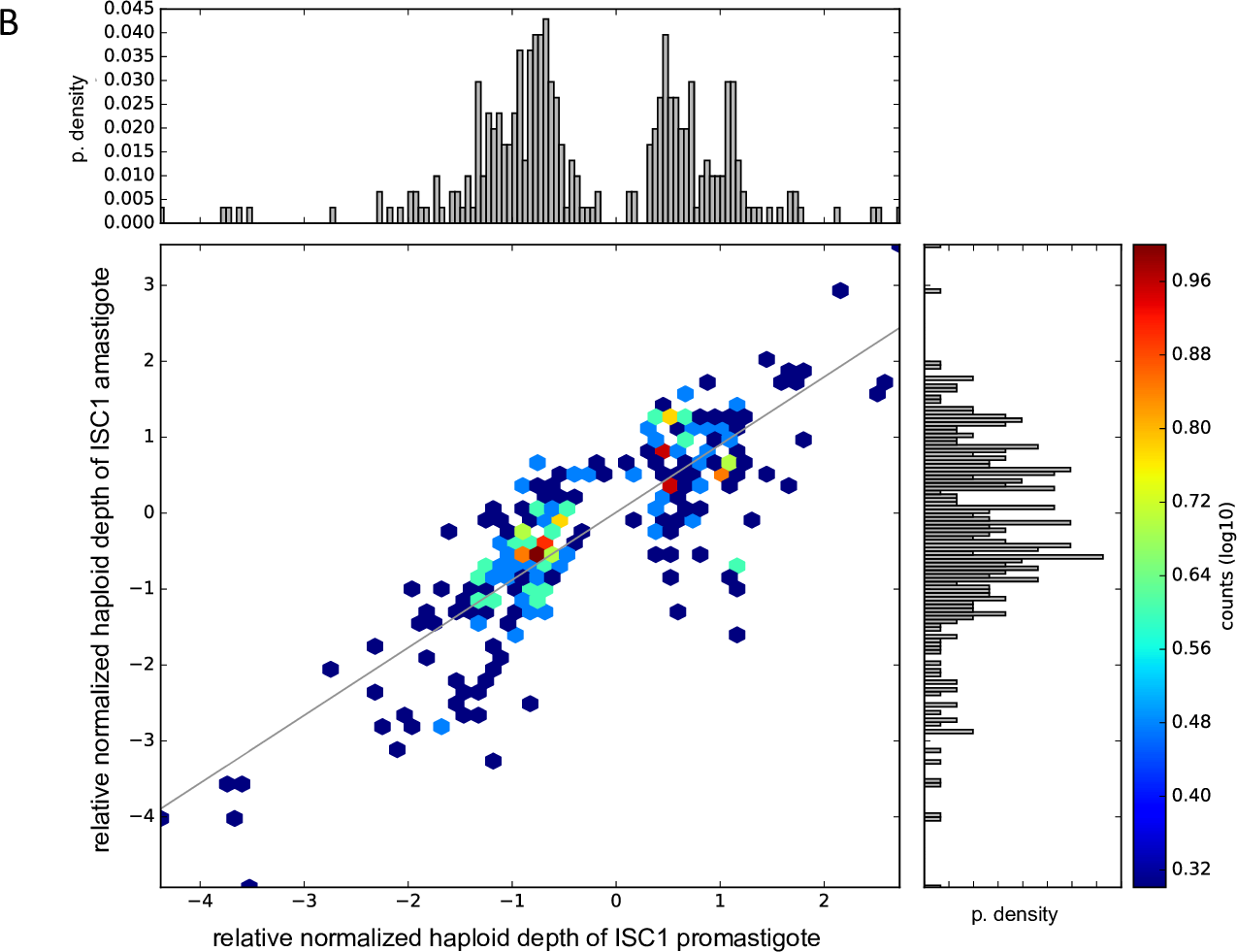

Supplement: S9 Fig — Correlation was moderately high r2 = 0.68, p-value = 10−76 and slope = 0.882 indicating that SuSL-seq can be used to confirm existing group-specific CNVs. On the plot, colored dots represent counts in log10 scale and probability density distributions were given on the corresponding axes. (TIF) [file pntd.0007900.s010.tif]

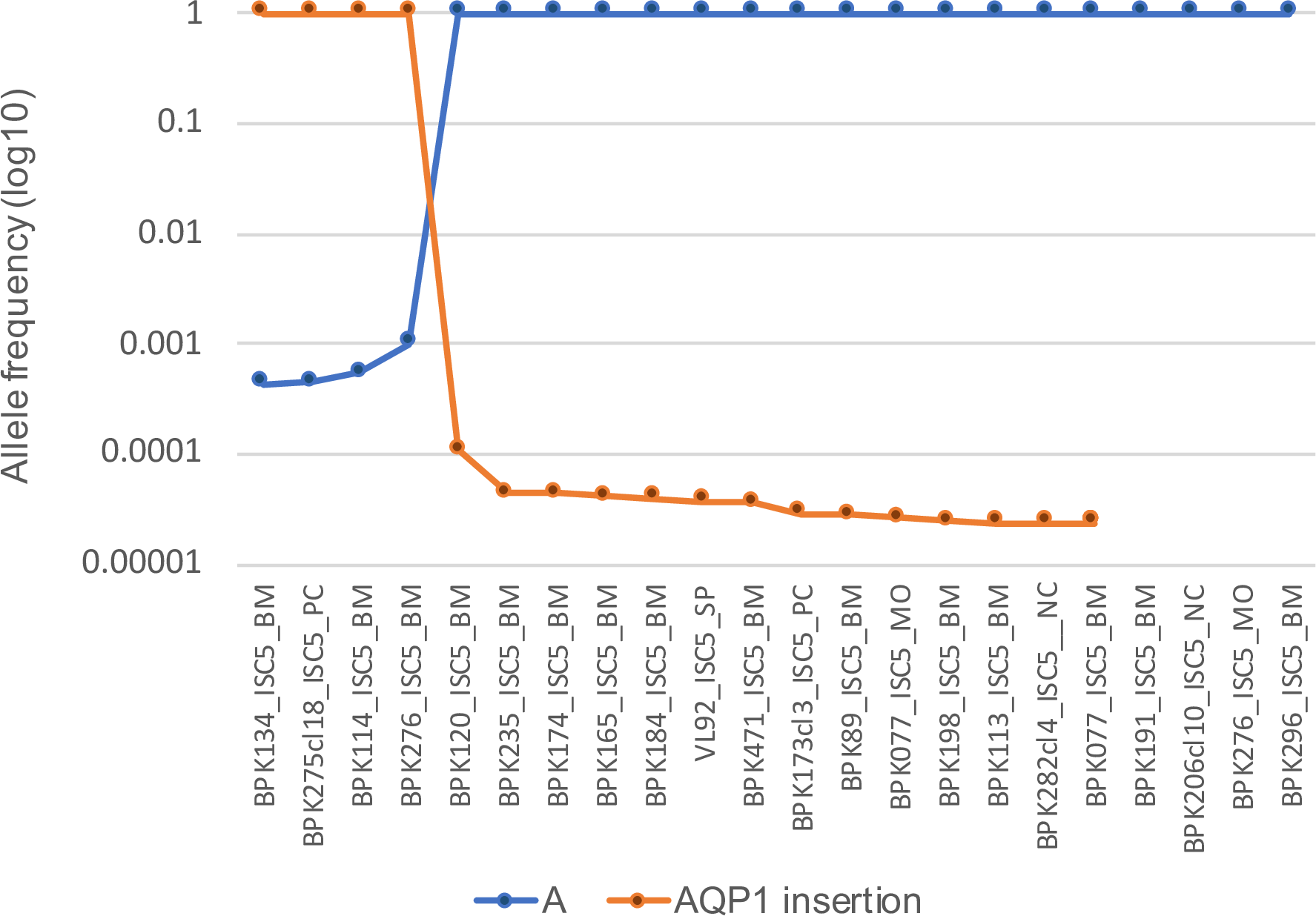

Supplement: S10 Fig — Blue line represents the frequency for the non-ISC5 allele, while the orange line represents the ISC5-specific allele, which contains a 2bp-insertion in the AQP1 locus. Arrows indicate the two clinical samples in which a second allele was detected above the background, being statistically significant for BPK120_ISC5_BM. BM, Bone Marrow; SP, Spleen aspirate; MO, cultured isolate; NC, Negative Control, a cloned strain where the diagnostic ISC5-specific allele is absent; PC, Positive Control, a cloned strain characterized by the presence of the diagnostic ISC5-specific allele. (TIF) [file pntd.0007900.s011.tif]

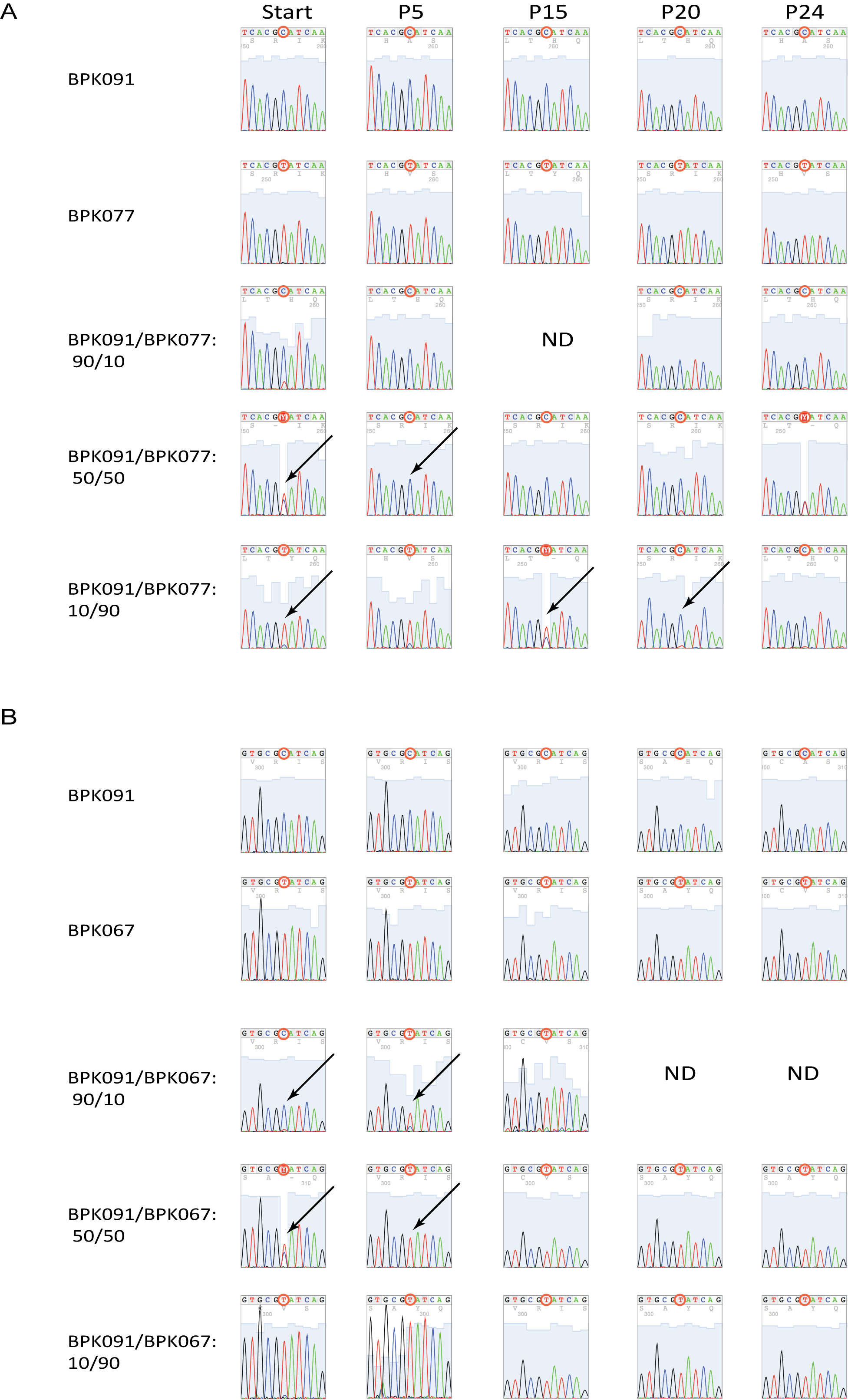

Supplement: S11 Fig — Flasks were inoculated with individual cloned strains or mixtures of two cloned strains in the following ratios: 90/10, 50/50 and 10/90. Cultures were analyzed at the beginning of the experiment (Start), after 5, 15, 20 and 24 passages (P5, P15, P20 and P24 respectively). Diagnostic PCR that allows to distinguish between the two compared strains was used to monitor the presence of each strain in culture. A selected fragment of chromatogram containing the variable nucleotide (labelled with red circle) is shown. Arrows point at the samples were the changes in dominance occurred. A. Competition between the BPK091, and BPK077 strains (ISC4, and ISC6, respectively). B. Competition between the BPK091, and BPK067 strains (ISC4, and ISC3, respectively). (TIF) [file pntd.0007900.s012.tif]
